# PPI Modulators of E6 as Potential Targeted Therapeutics for Cervical Cancer: Progress and Challenges in Targeting E6

**DOI:** 10.3390/molecules26103004

**Published:** 2021-05-18

**Authors:** Lennox Chitsike, Penelope J. Duerksen-Hughes

**Affiliations:** Department of Basic Sciences, Loma Linda University School of Medicine, 11021 Campus Street, 101 Alumni Hall, Loma Linda, CA 92354, USA; lchitsike@llu.edu

**Keywords:** protein–protein interactions, cervical cancer, HPV E6, small molecules, peptides, drug discovery, targeted therapy

## Abstract

Advanced cervical cancer is primarily managed using cytotoxic therapies, despite evidence of limited efficacy and known toxicity. There is a current lack of alternative therapeutics to treat the disease more effectively. As such, there have been more research endeavors to develop targeted therapies directed at oncogenic host cellular targets over the past 4 decades, but thus far, only marginal gains in survival have been realized. The E6 oncoprotein, a protein of human papillomavirus origin that functionally inactivates various cellular antitumor proteins through protein–protein interactions (PPIs), represents an alternative target and intriguing opportunity to identify novel and potentially effective therapies to treat cervical cancer. Published research has reported a number of peptide and small-molecule modulators targeting the PPIs of E6 in various cell-based models. However, the reported compounds have rarely been well characterized in animal or human subjects. This indicates that while notable progress has been made in targeting E6, more extensive research is needed to accelerate the optimization of leads. In this review, we summarize the current knowledge and understanding of specific E6 PPI inhibition, the progress and challenges being faced, and potential approaches that can be utilized to identify novel and potent PPI inhibitors for cervical cancer treatment.

## 1. Background

Cervical cancer (CC) is an oncologic disease of the uterine cervix caused by an oncogenic virus called human papillomavirus (HPV). According to WHO estimates, about 600,000 new cases are recorded annually, and more than half of these people die [1,2]. Finding ways to reduce this huge burden of cervical cancer is therefore imperative. The advent of anti-HPV prophylactic vaccines and their continued use will help with reducing the number of cases significantly in the future. For this approach to be effective, the vaccine must be administered pre-infection, and the rates of vaccine uptake need to be high. Unfortunately, vaccine coverage in both developed and developing countries has been suboptimal, with less than 50% coverage in most regions across the globe [3,4]. If these challenges persist, new cases of cervical cancer will remain significant, and the need for more effective clinical management of the disease will continue. Generally, when the disease is caught early, when surgery and/or radiotherapy can be used, the clinical outcomes are good with cure rates between 80% and 90% [5]. For stages IIB to IVA, a multidisciplinary approach that usually involves chemoradiation is employed. For patients with stage IVB or metastatic disease, systemic therapy is the standard of care. Even though recurrence is only between 10% and 20% for early stage CC, it can be as high as 50% to 70% for advanced disease within 2 years of completing treatment [6,7]. Once the cancer recurs, the 5-year survival rate is <5%. Patients in this recurrent, persistent, and metastatic category, therefore, represent a current clinical challenge and need that has not been met [7,8,9,10,11,12].

The standard of care (SOC) for patients with advanced CC is systemic therapy, and cisplatin has been the cornerstone of this treatment since its introduction in the early 1980s [8,9,10,11]. Given the limited efficacy in this patient subgroup, improving survival has always been an area of interest. It was initially observed that increasing the cisplatin dosage enhanced response rates (RRs) yet did very little to improve overall survival (OS) [8,9,11]. Furthermore, at these higher doses of cisplatin, toxicity was often intolerable. Other platinum-based agents, such as carboplatin, were less toxic but had lower response rates. Another issue that arose was platinum resistance [8,11]. To overcome some of these problems, several combinations of chemotherapeutics, where cisplatin-containing agents were paired with agents such as topotecan, gemcitabine, vinorelbine, were evaluated in various Gynecologic Oncology Group (GOG) trials. Of these many studies, GOG-169 in 1999 showed that the addition of cisplatin to paclitaxel doubled the RR and progression-free survival (PFS). A decade and half later, the GOG-240 trial successfully tested the addition of bevacizumab to the cisplatin–paclitaxel doublet, and this combination became the first combination study to demonstrate an improvement in OS (added 3 months) without a decline in quality of life. In 2014, this chemobiologic cocktail became the first-line choice of treatment for patients with advanced disease [8,9,10,11,13]. Because bevacizumab inhibits a specific target called VEGF, the study has a renewed interest in the idea that other targeted agents could offer additional gains in survival. Accordingly, a number of nonangiogenic cellular effectors were targeted in various clinical studies over the past few decades. Surprisingly, targeting the proliferative and prosurvival pathways in CC has been largely disappointing. Numerous EGFR inhibitors, including both monoclonal antibodies and small molecules, as well as mTOR analogues, have all failed to progress beyond phase III trials. Inhibitors of HDACs and PARP have also underperformed [9,10,11]. These findings and the fact that treatment regimens and OS have barely changed since the inception of cisplatin 4 decades ago highlight at least two points: (i) there exists a significant lack of clinically efficacious and selective agents, and (ii) there exists therefore a need to consider alternative targets or therapeutic strategies for CC.

One such alternative target are HPV oncoproteins. HPV oncoproteins are not only required for the initiation of cervical cancer, but also required for the maintenance of the disease. One of these viral proteins is E6, which affects an oncogenic phenotype through protein–protein interactions (PPIs). Indeed, studies have shown that E6 interacts with over 100 human cellular proteins [14]. A number of the host proteins targeted by E6 possess tumor suppressor properties, and genetic abrogation of E6 in primary cells or animal models induces growth arrest, followed by apoptosis or rapid senescence [15,16,17,18,19,20,21]. Importantly, combining this genetic manipulation with clinically approved cytotoxic agents potentiates the efficacy of those therapies [19,21]. E6 is therefore a potentially viable therapeutic target whose inhibition may yield clinical benefits, alone or in combination. Various studies, as discussed below, have been published recently with data on inhibitors that interfere with the interaction of E6 with host tumor-preventing proteins, raising the possibility of the PPIs of E6 as a possible target for pharmacological inhibition.

In this review, we provide a brief history of clinical management of CC and reveal the clinical needs that exist currently. We describe the important role of E6 in the maintenance of malignancy in CC through protein–protein interactions (PPIs) and highlight studies aimed at pharmacologically intervening in the specific PPIs of E6 to suggest that PPI inhibition is potentially a reasonable alternative anticancer approach. We then discuss the shortcomings of the studies and challenges the field faces, and give future perspectives that could further advance therapeutic development of E6 PPI modulators.

## 2. Protein–Protein Interactions of E6

E6 is a 150-amino acid protein comprising two intrinsically disordered termini and tandem repeat domains connected by a helix linker [22,23,24,25,26]. E6 is known to play a pivotal role in the life cycle of the HPV and numerous oncogenic processes involved in the initiation and maintenance of HPV-associated malignancies. Among these processes are cell survival, proliferation, antiapoptosis, differentiation, and metabolic reprogramming [27]. This multifunctionality of E6 stems from its ability to regulate the function of over 100 host cellular protein substrates [14,27,28,29]. An intriguing question then is, how does such a very small protein with no intrinsic enzymatic activity modulate the function of a multitude of foreign host proteins? The answer lies in the ability of E6 to engage in specific protein–protein interactions with many of these substrates, thereby hijacking their function through domain–motif interaction networks. The best characteristic of these networks is the E6–LxxLL interaction [22,23,24,25,26,30,31]. A charged, leucine-rich, alpha-helical motif, LxxLL, is found in a number of host proteins. One well-known example of this motif is the E3 ligase E6AP, which recognizes and binds to the hydrophobic pocket formed by the two zinc fingers and the linker helix of E6. The LxxLL motif stabilizes the conformation of E6, enabling the recruitment of the tumor suppressor p53 to bind to a cleft on E6. In this stable ternary complex, the E6AP ligase is catalytically activated and/or is close enough to tag p53 with ubiquitin molecules for degradation by the ubiquitin–proteasome system (UPS) [24,25]. This leaves the cell devoid of important functions of p53, such as apoptosis induction, cell cycle arrest, DNA damage sensing, and the suppression of tumorigenesis. In addition to E6AP, several other substrates carry the LxxLL motif, including MAML1, IRF3, paxillin, and so forth [22,25,26]. Another well-documented cellular function hijacking strategy by E6 involves PDZ domain proteins, such as MAGI-1, DLG1, hScrib, and 14-3-3ζ [32,33,34,35]. Some host proteins use short linear motifs (SLiMs) of 3–10 amino acids to modulate cellular function, and viruses such as HPV have evolved mimics of these SLiMs to usurp functions mediated by PDZ domain–motif networks [36]. Specifically, the C-terminus of E6 is unstructured and contains an x-T-x-L/V_COOH_ peptide motif known as the PDZ-binding motif (PBM). The E6 PBM acts as a competitive bait ligand for host PDZ domain proteins and helps to mark them for degradation via E6AP-dependent and independent mechanisms [37]. The PBM sequence is not found in the E6 of low-risk, non-cancer-causing HPV subtypes, and this indirectly reflects the importance of the E6 PBM in the development and progression of cervical cancer by regulating cancer-related processes, such as cell polarity, attachment, proliferation, and differentiation [35].

**Figure 1:**
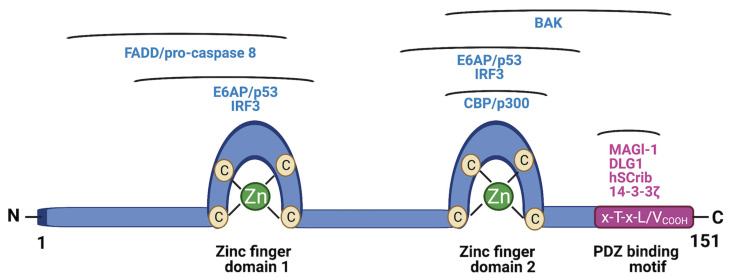
Schematic structure of HPV E6. Two zinc finger domains of E6 are shown, together with regions that are involved in interacting with the cellular substrates indicated above the regions. At the C-terminus is the PDZ-binding motif (PBM) and the associated PDZ proteins that bind to it. (Figure created using Biorender).

A number of key molecules in the apoptotic pathway are the subject of accelerated degradation mediated by E6. For instance, our lab has found that TNFR1, FADD, and caspase 8 are all substrates of E6. We demonstrated, through site-directed mutagenesis, that the terminal regions of these proteins are important in the binding to E6 [38,39,40,41]. We found that TNFR1 had an (E/D)L(L/V)G motif in the death domain (DD) in the C-terminus, and noted that this motif had been found to be important for binding to E6 in other substrates [42]. Even though variants of the L2G box, another motif common in E6 substrates, were found in the death effector domains (DEDs) of FADD and caspase 8, it was actually a novel motif further upstream in the N-terminus that was important in the binding of E6 [40,43]. The degradation of these three substrates in the presence of E6 compromises the extrinsic apoptotic signaling and facilitates cell survival. Two additional substrates of E6 are the coactivating transcriptional proteins CBP and p300. Studies show that E6 binds to three regions of both CBP and p300, namely, CH1, CH3, and the C-terminus. Consequently, it has been demonstrated that E6′s binding to CBP/p300 inhibits transactivation of p53 and NF*κ*B, and that the inactivation of these substrates causes a blockade on cell cycle arrest and senescence among other antiproliferative processes [44]. These well-known substrates of E6 and the pathways they are involved in are shown in Figure 1. Other substrates of E6 are not as well characterized in terms of their nature of interaction with E6 or their role in the oncogenesis of HPV-associated cancers as the ones mentioned above [14,28]. Also important to note is that while many do, not all interactions of E6 lead to degradation. Prime examples that fit this description include CBP/p300, hTERT, and IRF3. It is expected that future studies will uncover novel mechanisms, new E3 ligases, and other members of the UPS to shed more light on how E6′s interactions with some of these substrates drive cervical cancer development [14,27,28,45,46]. What is clear is that E6 binds to multiple targets and that specific perturbation of these PPIs has potential therapeutic utility in cervical cancer.

## 3. Therapeutic Targeting of E6 PPIs

Targeting the PPIs of E6 is a task with a fair share of challenges, but one that is by no means unfeasible. The first step towards determining the druggability of E6 and designing PPI modulators is the characterization of the interface of E6 PPIs in terms of the dynamics that govern the stability of binding of E6–ligand complexes and the residues that contribute most significantly to the free energy of binding (hot spot residues). This can be achieved through X-ray crystallography and alanine scanning experiments in conjunction with in silico tools, such as computational hot spot prediction and molecular dynamic simulations (MDSs). Fortunately, the 3D structures of E6 in complex with p53/E6AP and PDZ proteins are now available, as are several site-directed mutagenesis studies [22,25,26]. These studies reveal that E6 has a hydrophobic pocket that recognizes the LxLL motif of E6AP. Moreover, the residues of E6 that make hydrophobic and polar contacts with the hot residues of E6AP and the intermolecular interactions at the interface of E6 and p53 are now also well known. This structural knowledge has provided a framework for designing inhibitors that stop E6 from binding to several of the host proteins mentioned above, such as E6AP, p53, caspase 8, p300, and PDZ-domain containing factors. These studies are summarized in the following sections.

### 3.1. E6–E6AP Interaction Inhibitors

The evolution of designed inhibitors that target the binding interface of E6 with E6AP started with peptide-based inhibition almost 2 decades ago. With insights from the solution structure of the E6-interacting peptide of E6AP and mutagenesis manipulation, the design began with 18 amino acid peptide sequences spanning the LxxLL motif as competitive antagonists. Most peptides were made without stabilization modifications. Importantly, these peptide derivatives were able to competitively inhibit E6AP binding to E6, and these findings became the first crucial step towards the dream of targeting E6 in HPV-associated cancers [47,48,49,50,51,52]. The obvious drawback these peptides had, however, was their relative weak affinity and lack of robust activity in HPV^+^ cancer cell lines. In addition to peptides, proteins, protein scaffolds, and intracellular antibody fragments were successfully used to specifically inhibit E6 and thus prevent it from binding to its substrates. For instance, expressions of pit2a, intrabodies, and peptide aptamers in HPV^+^ cells have been demonstrated to stabilize p53 and decrease cell viability [53,54,55,56,57]. Building on the aforementioned research on proteins and peptide-based inhibitors, two labs independently combined these concepts to develop similar novel inhibitors that bind E6 in a multivalent fashion [58,59]. Specifically, these protein-based inhibitors composed an LxxLL peptide motif linked to a PDZ domain. These bivalent inhibitors were shown to interact with E6 both intracellularly and in vitro by binding to the helix pocket and the PBM in the C-terminus of E6. One notable advantage these new inhibitors had over the previous generation of peptides or proteins was their strong affinity for E6 binding. The K_d_ of the individual PDZ bodies and LxxLL-based peptides used in these two studies were determined to be in the lower micromolar ranges. In contrast, the bivalent inhibitors demonstrated nearly a 1000-fold increase in their binding affinities, with K_d_ values of 10 and 65 nM, respectively [58,59]. These studies show that mimicking the native ligands and domains recognized by E6 can be an effective way to inhibit E6. Interestingly, a study by Dymalla et al. showed that E6 can also be targeted by peptide ligands with no resemblance to the canonical LxxLL motif [60]. In their study, they screened a randomized peptide expression library for E6 inhibitors and identified a peptide with a novel motif. Further studies revealed that a solubility-optimized variant of this peptide containing 19 amino acids (pep11**) was able to bind to the same hydrophobic groove where the E6AP LxxLL peptide binds. Not only did this peptide displace E6AP both in vitro and in vivo with a higher affinity than the E6AP-LxxLL peptide, but also its expression in vivo was able to rescue p53 and caused apoptosis in HPV^+^ cells [22,23,60]. These improvements notwithstanding, pep11**, like the other peptide-based ligands, still had to be delivered to the cells via an expression vector due to cell permeability limitations.

Such limitations of peptide-based inhibitors of the E6–E6AP interaction have inspired the quest to find small organic molecules as alternatives. One of the first groups to rationally adopt this approach was the Androphy lab around 2006 [61]. They began by creating a pharmacophore model based on the LxxLL peptide ligand. With that, they queried large compound databases and then characterized the resulting candidate inhibitors of the E6–E6AP interaction. Their efforts identified a number of novel compounds with appreciable in vitro activity and selectivity, such as compound 9. One limitation was that the study did not provide experimental validation of in vivo activity in cell-based assays [61]. Nevertheless, the study provided sound proof of principle and served as a basis for future investigations. Indeed, a follow-up on this study using a similar approach was later carried out using an optimized pharmacophore model. This led to the discovery of luteolin, a flavonoid compound. Characterization using E6–E6AP filter-based in vitro competition binding, structure–activity relationships (SARs), and cell viability assay confirmed its anti-E6 activity and showed that luteolin and a number of its analogs were capable of reconstituting p53 and inducing apoptosis [62]. Molecular modeling and docking studies in a different study further authenticated that, indeed, these compounds were binding to the hydrophobic LxxLL groove, and identified the substituents important in the interactions [63]. Not long after, another group published results identifying several additional flavonoid compounds with the ability to inhibit E6–E6AP. Using an ELISA-based E6–E6AP high-throughput screening assay, Malecka et al. found that compounds such as gossypetin and baicalein were capable of inhibiting E6AP from binding in vitro. In cells, these compounds stabilized p53 and induced apoptosis in E6-expressing cells [64].

In addition to in vitro binding screening assays, in silico methods have also been used to identify E6–E6AP inhibitors. An example is the recently published Ricci-López et al. study. Using 26 reference compounds, mostly flavonoids, known to inhibit E6, this group created a large compound library based on structural similarity to the references, and then utilized docking screening and molecular dynamic simulations to virtually screen and validate the hits. They identified three novel compounds, lig1, 2, and 3 [65]. However, follow-up experiments to validate p53 rescue or apoptosis induction in HPV^+^ cells are still pending.

### 3.2. E6–p53 Interaction Inhibitors

Rather than focusing on preventing the binding of E6 to E6AP, a prerequisite for p53 ubiquitination and degradation, other groups have looked for inhibitors that directly prevent interactions between E6 and p53. One such inhibitor is called jaceosidin, another flavonoid. Lee et al. isolated jaceosidin and confirmed its ability to interfere with E6–p53 association using an ELISA-based in vitro binding assay. They also showed that this small molecule reduced the viability of the two cervical cancer cell lines, CaSki and SiHa [66]. In 2010, Zhao et al. also showed that this approach can be effective when they demonstrated through pull-down assays that a molecule called RITA can free p53 and restore its transcription and proapoptotic function. More importantly, RITA inhibited HeLa cell growth in a xenograft mouse model [67]. In silico approaches have also been used to identify E6–p53 inhibitors. Shaikh et al. docked about 88 small molecules for molecular screening and identified nicandrenone as a potential inhibitor of E6–p53 [68]. Even though there was no experimental wet-lab validation in this study, a different group recently published a larger in silico study with experimental validation. Using the crystal structure of E6–E6AP–p53, Celegato et al. virtually screened a library of almost a million compounds. They discovered a hit, compound 12, and authenticated its binding in vitro using an ELISA-based E6–p53 binding assay. In addition to restoring p53 and its transcriptional activity, compound 12 inhibited cell viability and 3D cervosphere formation [69].

### 3.3. E6–Caspase 8 Interaction Inhibitors

As with E6–E6AP targeting, the targeting of the E6–caspase 8 inhibition also began with peptide-based inhibitors. Following the identification of regions in the death effector domains (DEDs) of FADD and caspase 8 that are important in the binding of E6 via site-directed mutants and pull-down assays, our lab developed an AlphaScreen^TM^ assay to assess competitive binding. We demonstrated that, indeed, the 23-mer and 16-mer peptides of FADD and caspase 8, respectively, were capable of antagonizing E6 binding to the respective substrates. Functionally, the expression of these peptides in cervical cancer cells resensitized cells to apoptotic stimuli induced by TNF and Fas ligands [38,39,40,41]. These studies became the foundation for screening small-molecule libraries in search of E6-specific pharmacological inhibitors. Using FADD and caspase 8 as substrates, we identified a number of flavonoid compounds, including myricetin, morin, quercetin, and kaempferol [70]. We followed up on these findings in search of additional E6 inhibitors by screening a second library, which resulted in the identification of a novel inhibitor with an imidazole-derived scaffold called spinacine. We demonstrated that both myricetin and spinacine were capable of rescuing caspase 8 and p53 function and enhancing the efficacy of various apoptosis-inducing agents, such as cisplatin, doxorubicin, and TRAIL in HPV^+^ but not HPV^-^ cell lines [71]. Further characterization of myricetin, particularly its binding interactions, has been carried out computationally. Using molecular modeling, it was found that myricetin engages amino acid residues such as Leu50 and Cys51 that are found deep in the alpha-helical groove, and this may help to explain its ability to inhibit E6–E6AP interactions [72]. In addition to these inhibitors, we recently discovered another inhibitor with a nonflavonoid scaffold (30 hydroxygambogic acid, or GA-OH) as a potential therapeutic for HPV^+^ cancer cells. We demonstrated that GA-OH significantly inhibited the survival and growth of HPV^+^ cell lines and displayed higher potency than did flavonols such as myricetin [73]. In the future, we hope to study whether this inhibitor will potentiate the activity of standard therapies in HPV-associated cells and how it interacts with E6.

### 3.4. Other Interaction Inhibitors

**Figure 2:**
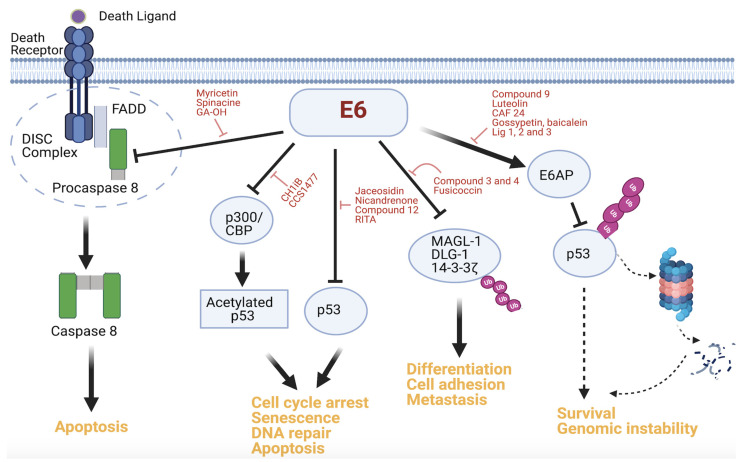
Actionable PPIs of E6. Host protein substrates that interact with E6 and the affected downstream effectors and oncogenic signaling are shown. The small-molecule inhibitors that perturb the binding of E6 to these substrates have been identified and are highlighted. The processes that are impacted by these modulators include apoptosis, cell cycle arrest, differentiation, and metastasis. (Figure created using Biorender).

As indicated above, E6 has a multifaceted inhibitory capability against p53, and can suppress p53 activity directly through E6AP or indirectly through activators of p53 such as p300 and ADA3 [44,74]. Exploiting this alternative strategy by E6, Xie et al. showed that the CH1 domain of p300 can competitively inhibit E6 binding to p300 in cells. This effect was associated with p53 functional reactivation and apoptosis induction and inhibition of the growth of HPV^+^ tumor cells in a NOD/SCID mouse model. Treating cells with a novel CH1iB small molecule, a synthetic mimic of the HIF1 α-helix, recapitulated these phenotypic effects and potentiated the efficacy of cisplatin in HPV^+^ cells [75]. Consequently, agents that target the CH1 domain are garnering more interest as antiproliferative cancer treatments. Novel orally available small-molecule ligands that are directed at the CH1 domain of p300/CBP have been discovered. One of these small molecules, CSS1477, has shown antitumor activity in animal models, including HPV-associated tumor xenografts, and its prospects for phase I clinical trials are considered high (ASCO, ACCR, Cell). Besides the E6–p300 association, another PPI that has been less extensively targeted is the interaction between E6 and the PDZ proteins. Nonetheless, Tian et al. designed pharmacophores based on the E6 PDZ-binding motif (PBM)–PDZ complex and used the resulting pharmacophores to screen a commercial compound database. They discovered two promising candidate compounds (compounds 3 and 4) with this approach, although experimental validation of the ligands has not been reported [76]. A recent preprint article also investigated the potential druggability of the E6 PBM by targeting the E6–14-3-3ζ interaction. The study shows that the two proteins interact and that a small-molecule stabilizer of 14-3-3ζ, fusicoccin, weakens the E6–14-3-3ζ interaction using polarization and X-ray crystallography [77]. Figure 2 gives an overview of the inhibition of all the aforementioned PPIs of E6.

## 4. Progress and Limitations of Current E6 PPI Studies

Only a little over 20 years ago, PPIs were considered “undruggable” and for good reason. Unlike traditional enzymes, ion channels, and receptors that bind to small well-defined pockets, PPIs tend to occur at interfaces with large, featureless, and sometimes noncontiguous surfaces [78]. Nevertheless, the notion of intractability has been changing as molecules that perturb PPIs continue to emerge with some already in clinical use [79,80,81,82]. For E6, like many other PPI proteins, the evolution of inhibitors began with the availability of high-resolution structures of E6 that then informed the design and in silico analyses of E6–ligand interactions. From these atomic structures, we learned that the buried surface area at the interface with substrates such as E6AP and p53 is only between 900 and 1200 Å^2^ [25,63]. This surface area falls into the category of “narrow” PPI interfaces (<2500 Å^2^), which generally has been found to be amenable to inhibition with small-molecule ligands [83]. This prediction of druggability has been supported by the occupation of similar binding spaces by both natural binding partners of E6 and small-molecule ligands. Specifically, molecular docking and MDS have demonstrated that ligands bind in the same space that recognizes the LxxLL motif, and that there is overlap of interactions of residues between ligands and natural binders, such as E6AP, p53, and caspase 8 [22,63,72,84,85]. In addition, these studies have revealed the importance of the positive polar patches from residues such as arginines forming the rim of the pockets and some details of how they stabilize E6 complexes. In addition to docking and simulations, other strategies for identifying E6 PPI modulators, such as high-throughput screening (HTS) techniques, fluorescence polarization, ELISAs, and AlphaScreen^TM^ technology, have played critical roles in uncovering or validating both chemical ligand- and peptide-based inhibitors. A summary of the prominent E6-specific inhibitors reported thus far and the endpoints assessed is shown in Table 1. As evident in this scheme and the discussions above, the majority of the small-molecule inhibitors of E6 found so far are natural compounds, mostly flavonoids. Interaction studies have shown that the aromatic rings on flavonoids match favorably with the E6 helix groove, as their carbons interact with hydrophobic residues. The free hydroxyl groups or carboxylic groups common in these ligands are involved in stabilizing polar intermolecular interactions. As such, removal of these polar substituents was associated with a decrease in activity [62,85]. These revelations may explain the dominance of flavonoid compounds as E6 inhibitors thus far.

Despite this progress in understanding E6 and inhibiting its PPIs, significant gaps in our knowledge remain, and significant strides are still needed to match previous successes in inhibiting the PPI of other proteins [80,81]. First, the ligands discovered thus far are generally not high-affinity binders, and their potency in cells is fairly low, usually in the low to mid micromolar ranges [61,62,63,64,66,69,70,71]. Not surprisingly, the majority of these studies have failed to progress to animal studies or clinical trials. A second observation is that there is a general lack of chemotype diversity in the collection of E6-inhibiting small molecules, as most compounds are natural products with a flavonoid chemotype. Not only have natural compounds been generally de-emphasized for drug development, but they also tend to be difficult to characterize, synthesize, and chemically modify. Flavonoid compounds have a number of chemical and metabolic liabilities in terms of synthetic tractability, aqueous solubility, permeability, specificity, and bioavailability [86,87,88,89]. The same can be said about the peptide-based inhibitors that make up a fair share of E6 PPIs, as they often suffer from permeability and bioavailability issues. Therefore, improving the properties of discovered leads, as well as the development of novel, high-affinity, and selective binders with good physicochemical properties, is imperative.

## 5. Conclusions and Future Perspectives

The past 2 decades has seen several technological breakthroughs and novel ways to answer questions surrounding PPIs, and tremendous progress has been made even for PPIs initially deemed difficult to target pharmacologically. Research on PPIs of E6 is progressing, but is still comparatively limited in scope and in the strategies employed to more effectively target the interacting interfaces. One way to start to address some of the challenges presented earlier is to leverage the natural products available to derivatize new leads. The topological makeup of natural products makes them ideal templates for new ligands with suitable scaffolds for inhibiting PPIs [83]. Using organic chemistry breakthroughs such as scaffold hopping, rescaffolding, substitution, and isosteric replacement reactions could change the topology or the functional groups of natural compounds to create entities with better druglike properties [90,91]. For instance, the derivation of vadimezan, now a clinical agent, which possesses a xanthenone scaffold from a flavonoid compound, showcases how structural modification through hopping can improve the potency of existing leads and their PK properties [91]. In the same vein, efforts to inhibit E6 PPIs could benefit from chemically more diverse libraries. New synthetic strategies that expand the chemical space of PPI inhibitors by building libraries with better chemical diversity and structural complexity now exist through synthetic methods such as diversity-oriented synthesis (DOS) and multicomponent reactions (MCRs) [92]. The same can be said for peptides, another type of PPI modulator that has been used to inhibit E6. Significant advances in chemistry can now overcome some of the associated drawbacks, such as typically low bioavailability. The use of unnatural amino acids, retropeptides, bioisosteric modifications, and macrocyclization- and hydrocarbon-stapled peptides has improved many of the traditional drawbacks [93]. The CH1iB peptide mimic described above was designed using a similar approach called hydrogen bond surrogate strategy, and it showed good activity when used to treat cells [75]. In addition, small-molecule-based scaffolds can be used to create nonpeptide structural and functional mimics [94]. For instance, Beceril et al. successfully created a pyridylpyridone α-helix mimetic that competed with natural LxxLL peptide ligands of coactivators for binding estrogen receptors (ERs) [95].

To find new and novel classes of E6 PPI inhibitors, it will also be necessary to explore new strategies for studying and probing PPIs. Most known E6 PPI inhibitors were discovered using HTS techniques and, to a lesser extent, virtual screening. HTS has certainly been successful at identifying PPI inhibitors. However, other techniques have been found to be better. Fragment-based drug discovery (FBDD) is one such technique, with a higher hit rate and ligand efficiency for PPIs that are especially difficult to target [78]. In FBDD, simple low-molecular fragments from a fragment library are screened using biophysical or biochemical methods for molecules that bind to regions most critical for PPIs. Once the fragment hits (often of low affinity) have been identified, sensitive biophysical methods are often used to determine the binding mode of ligands. Using this information, a structure-based drug design can then be used to rationally and systematically grow the fragment [96,97,98]. A similar approach has also been used for PPI inhibitors specifically aimed at creating chemotypes that mimic the critical residues in the interface. In this approach, an anchor residue has to be established (none has been found yet for E6) from the hot spot amino acids, and this then is used for substructure search and bioisosteric design. Once initial leads are available, crystal structures of the protein–ligand complexes are created and systematic optimization through medicinal chemistry is carried out [78]. Such methodical, rational, and repetitive efforts to optimize identified hit compounds into leads with favorable physicochemical, PK, and in vivo properties have not yet been done in the context of inhibitors of E6 PPIs. For most inhibitors found through HTS, there is often no follow-up work to analyze the binding mode and how substituents are interacting with residues in the pocket so as to leverage this information for structure-guided design and reiterative hit-to-lead optimization. Evidence in the field shows that a lack of such a collaborative approach in studying PPIs is likely detrimental to the hopes of effectively inhibiting PPIs, as discovery of the leads that have been approved or are in clinical trials has historically come through multidisciplinary work. One case in point is provided by a recent study by Dawidowski et al., which illustrates the type of work needed to improve the affinity and potency (by severalfold) of an initial hit into the final lead that inhibits the PEX5–PEX14 interaction. This study shows an interplay of structure-based virtual screening, NMR analyses, AlphaScreen-based competition assays, biochemical assays, X-ray cocrystals, and cycles of medicinal chemistry optimization [99]. It remains to be seen whether similar approaches will be utilized for studying E6 PPIs in the future. Importantly, it is crucial to keep in mind that barriers to utilizing some of the biophysical methods used to study PPIs are huge; not only is the equipment utilized in structural biology studies expensive, but also the techniques themselves and the data analysis require special expertise with steep learning curves. Additionally, the lack of anchor hot residues, the huge flexibility of residues such as rim arginines, and the biological degeneracy of E6 may present challenges that some of these proposed strategies cannot overcome. Nevertheless, it will be worthwhile to see how some of these novel approaches can change the fortunes of E6 PPI inhibition.

## Figures and Tables

**Table 1:** Summary of compounds tested for their ability to inhibit E6 PPIs.

PPI Targeted	Name of Inhibitor	Study Systems Utilized (In Silico, In Vitro, Cells, Animals)	Reported Potency (IC_50_/EC_50_)	References
E6–E6AP	Compound 9	E6–E6AP filter plates	17 µM (in vitro)	[61]
	Luteolin	E6–E6AP filter plates, CC cell lines	23 µM (in vitro)	[62,63]
	CAF-24	E6–E6AP filter plates, CC cell lines	5.2 µM (in vitro)	[62,63]
	Gossypetin	E6–E6AP ELISA, PA-E6 cell line	170 nM (in vitro)	[64]
	Lig1, 2, 3	E6–E6AP (in silico)	N/A	[65]
E6–p53	Jaceosidin	E6–p53 ELISA, CC cell lines	N/A	[66]
	RITA	Pull-down, CC cell lines, xenograft	N/A	[67]
	Nicandrenone	E6–p53 (in silico)	N/A	[68]
	Compound 12	E6–p53 in silico and ELISA, CC cells	12–27 µM CC cells	[69]
E6–procaspase 8	Myricetin	E6–Cas 8 AlphaScreen, CC cells	0.6–0.9 µM (in vitro)	[70,71]
	Spinacine	E6–Cas 8 AlphaScreen, CC cells	2 µM (in vitro)	[71]
	GA-OH	E6-Cas 8 AlphaScreen, CC, and HNSCC * cells	N/A	[72]
E6–p300	CH1iB	IP*, HNSCC* cells, xenograft	N/A	[75]
	CSS1477	HNSCC cells, PD* xenograft	N/A	[ASCO]
E6–PDZ domain	Compounds 3, 4	E6–PDZ (in silico)	N/A	[76]
	Fusicoccin	E6–14-3-3ζ X-ray, fluorescence polarization	N/A	[77]

* PD xenograft, patient-derived xenograft; HNSCC, head and neck squamous cell carcinoma; IP, immunoprecipitation.

## Data Availability

All materials are provided in this article.
